# Context-dependent fitness benefits of antibiotic resistance mutations

**DOI:** 10.1098/rspb.2024.1071

**Published:** 2024-07-24

**Authors:** Aabeer Basu, Laasya Samhita

**Affiliations:** ^1^ Ashoka University, Rajiv Gandhi Education City, Sonipat 131029, Haryana, India

**Keywords:** antibiotic resistance, genotype, environment, fitness cost, media-specific effects

Discordance between organismal genotype and phenotype is one of the fundamental curiosities in evolutionary biology [1]. The expression of a trait is determined not only by its genotype but also by the environment. As a result, organisms with the same genotype may express a phenotype to different degrees under different environmental conditions, generating a range of phenotypes [2,3]. Genotype × environment (G × E) interactions are known to affect a wide range of organismal traits and functions [4,5]. While well understood conceptually, such effects are rarely tested explicitly in experimental work, partly no doubt owing to operational constraints. On the contrary, experimental results are often generalized based on specific model organisms and conditions. Recent work suggests that our understanding of antibiotic resistance may be especially susceptible to this practice. Antibiotics, once heralded as miracle drugs, are rapidly running out of their usefulness. New resistance is reported frequently, and fresh drugs are not being produced at the same rate; as such, a future bereft of antimicrobial drugs is no longer a far-fetched dystopia [6]. Studying the evolution, dissemination and mitigation of antimicrobial resistance (AMR), therefore, is essential both in and beyond clinical settings. In order to do so, one must first be able to measure AMR accurately, as well as compare it across pathogens, often in different contexts and environments. A recent study by Soley *et al*. [7] warns us that this might not be an easy task.

The authors used rifampicin, a commonly used antibiotic and a frontline drug for tuberculosis treatment [8]. They isolated 11 spontaneous rifampicin-resistant *Escherichia coli* mutants, all with different amino acid substitutions. All mutations were located within a single gene (*rpoB*) that encodes the β-subunit of the RNA polymerase enzyme, the known target for rifampicin. Previous studies have shown that mutations in *rpoB* are necessary and sufficient to confer bacterial resistance to rifampicin across different bacterial species including *E. coli* and *Mycobacterium* spp. [9–11]. The key finding is that the degree of resistance to rifampicin varied depending upon assay medium. Strikingly, the relative resistance or rank order of mutants also changed, i.e. mutations that conferred the maximum resistance in LB medium no longer conferred the maximum resistance in other media, and the minimum inhibitory concentration (MIC) changed with the culture environment. While the results highlight variability in defining the degree of AMR, it is important to interpret them carefully. The rank order is generated by comparing areas under the curve from independent growth measurements, and may not be entirely representative of competitive fitness in a mixed environment. More importantly, the use of MIC as a necessary and sufficient parameter to define resistance has been debated previously [12–14], for its inability to capture other aspects of bacterial growth in antibiotics [15,16]. A study preceding the current one tested eight antibiotics in different growth media and found that the MIC was a fluid parameter even in the absence of any resistance mutations, an observation held out by models using growth rate differences across different carbon sources [17]. Studies highlighting G × E interaction in AMR are only now emerging. In contrast, both positive and negative epistatic interactions between antibiotic resistance mutations (G × G interactions) and their interplay with antibiotic concentrations are well explored [18]. For example, bacteria carrying point mutations that lead to streptomycin resistance (e.g. RpsL K43R and K88E) show fitness compensation upon acquiring specific second site mutations (e.g. GyrA D87G and RpoB D516V) in a genetic background-dependent manner [19]. The latter mutations also confer resistance to different classes of antibiotics, fluoroquinolones and rifampicin, respectively. The strength and direction of such interactions are modulated by at least one environmental parameter: that of drug concentration [20]. Altogether, culture-based estimation of AMR phenotypes is likely to be more complex than reading out a single MIC value.

Similar concerns can be raised with respect to the growing use of genome-based AMR predictions [21–23]. The goal is generally to detect AMR genes in isolated genomes or metagenomes sampled from environmental DNA and, with sufficient data, shed light on the emergence and spread of AMR in a given setting [24]. Metagenomic predictions rely on previously documented correlations between bacteria harbouring specific AMR elements and their documented phenotypes, such as MIC. Since such phenotyping is carried out in clinical settings, both the culture medium in the diagnostic laboratory and the micro-environment within the patient are likely to influence the outcome. How then can AMR phenotypes in the clinic be best assessed to recommend treatment? Even if the use of multiple culture media and conditions is built into standard diagnostic procedures, it is unclear which one should be considered the most representative for treatment. Synthetic media that mimic host body conditions could be one way forward and already constitute an area of intense research [25,26]. Another path taken currently is to factor in possible mismatches in assessing AMR, and use evolution-informed therapeutic strategies.

Evolution-informed therapeutic strategies aim to prevent the evolution of AMR using prior knowledge of collateral sensitivity and cost of resistance [27]. Collateral sensitivity describes a situation where a bacterial strain by virtue of having evolved or acquired resistance to one antibiotic becomes susceptible to another [28]. The results of Soley *et al*. [7] show that at least for rifampicin-resistant mutants, the occurrence of collateral sensitivity and resistance as well as the concomitant costs manifest in an environment-dependent manner. Resistant bacteria often (but not always) incur fitness costs and are expected to be out-competed by their susceptible counterparts in an antibiotic-free environment [29]. *Combination therapy*, where different classes of antibiotics are administered simultaneously, and *sequential therapy*, where different classes of antibiotics are administered in succession one after another, rely on collateral sensitivity to hinder the evolution of resistance to one antibiotic, and on resistance costs to prevent simultaneous evolution of resistance to all antibiotics [30–32]. Similarly, *intermittent administration of a single antibiotic* builds on an understanding of resistance costs: susceptible strains are expected to outcompete the resistant strains during the pauses and then be killed during the next round of drug administration [33]. It is, therefore, plausible that the predicted efficiency of evolution-informed therapies will differ even within the labratory depending upon the choice of media [34,35]. The therapies described above have so far met with mixed success in the clinic [36,37]. Although most antibiotic resistance mutations confer a fitness cost [38], cost compensation can be rapid and resistance costs among clinical isolates vary considerably [39], confounding predictions for treatment. In addition, the success of these therapies could differ by an even greater degree between individual patients since each patient is in a unique environment with variations in facets of immunity, microbiota and other epigenetic factors.

The idea that AMR phenotypes may be impacted by internal and external environments is not new; growth rate, metabolic differences and even nutrients used in culture [17,40] are known to impact the manifestation of AMR phenotypes. The laboratory study by Soley *et al.* [7], however, serves as a valuable reminder that AMR phenotypes must be recorded and interpreted with caution. Although the AMR literature is dominated by mutation-based resistance, several key clinically relevant AMR phenotypes, such as biofilm formation [41] and persistence [42,43], rely on transcriptional alterations. Soley *et al.* used *E. coli* with mutations in *rpoB*, a global transcriptional regulator, as their model system. As such, the presence of distinct mutant-specific transcriptional profiles is perhaps not surprising. The occurrence of media-specific transcriptomes suggests that the phenotypic landscape for AMR may be even broader than is currently estimated. Taken together, the study emphasizes that the environment is a major factor that determines both expression of AMR genes and the strength of the AMR phenotype across multiple antibiotics, even when the relevant mutations are within a single gene. Given that individual mutant fitness varies so much across media, could the culture environment also influence the selection of specific mutations, and therefore lead to alternative evolutionary paths across different media? Prior studies have noted differences in resistance frequency with a change in culture medium [40]. Community diversity influences the development and spread of resistance via horizontal gene transfer [44], while population size and mutation frequency are core determinants of any AMR evolution. However, to the best of our knowledge, we do not yet have clear evidence to suggest that environmental influences alter mutational paths in antibiotic-treated bacterial populations, opening up an exciting avenue for future exploration.

The role of the environment, and especially the potential for genotype-by-environment interactions, is only recently emerging in the field of AMR. The current study is an incremental step towards empirical exploration of this lacuna. It also cautions against generalization of AMR phenotypes, and has important implications for all facets of AMR mitigation.

## Data Availability

This article has no additional data.
